# Analysis of the Influence of *Lactiplantibacillus plantarum* and *Lacticaseibacillus rhamnosus* Strains on Changes in the Hexachlorobenzene Content in Fermented Mare Milk during Refrigerated Storage

**DOI:** 10.3390/molecules29020528

**Published:** 2024-01-21

**Authors:** Agata Witczak, Anna Mituniewicz-Małek, Izabela Dmytrów

**Affiliations:** Department of Toxicology, Dairy Technology and Food Storage, Faculty of Food Sciences and Fisheries, West Pomeranian University of Technology, 70-310 Szczecin, Poland; anna.mituniewicz-malek@zut.edu.pl (A.M.-M.); izabela.dmytrow@zut.edu.pl (I.D.)

**Keywords:** mare milk, probiotic bacteria strains, hexachlorobenzene, refrigerated storage

## Abstract

(1) Background: Hexachlorobenzene (HCB) is a persistent organic pollutant that is possibly carcinogenic to humans. It is still found in the environment, humans and animals, and in foods, including milk and dairy products; (2) Methods: The influence of the probiotic cultures *Lacticaseibacillus rhamnosus LCR* and *Lactiplantibacillus plantarum* subsp. *plantarum LP* on the possibility of effecting the biodegradation of HCB in dairy products fermented from mare milk was investigated, taking into account the product storage time (maximum 21 days). HCB content was determined using the GC/MS method; (3) Results: A strong negative Pearson correlation (*p* < 0.05) was found between HCB concentration and the refrigeration storage time of the fermented beverages. The highest HCB reduction was observed in milk fermented with both *Lacticaseibacillus rhamnosus LCR* and *Lactiplantibacillus plantarum subsp. plantarum LP* (78.77%), while the lowest was noted when only *Lactiplantibacillus plantarum subsp. plantarum LP* was used (73.79%); (4) Conclusions: This pilot study confirmed that probiotics commonly used to give products health-promoting properties can also contribute to reducing the content of undesirable substances, and the bacterial cultures used might provide an alternative method for reducing HCB residues in fermented drinks.

## 1. Introduction

Hexachlorobenzene (HCB) has been used in agriculture as a pesticide and fungicide [1] and as a chemical intermediate in many industrial processes, such as in the rubber industry, in the production of dyes, and as a flux in aluminum smelting. Metallurgical processes, including aluminum production, steelmaking through electrolysis, copper refining and metallurgy, have been among the main sources of HCB in the environment [2], as have tire incineration plants, HCB treatment plants, and chlorine bleaching in the paper industry [3]. HCB has also been used in pyrotechnics for military purposes, and as a porosity regulator in the production of electrodes [4].

Currently, HCB is produced as a by-product in some industrial and technological processes when temperatures exceed 200 °C and the raw materials contain organic compounds and chlorine or its derivatives. Significant amounts of HCB are also produced as waste in the production of chlorinated solvents, such as perchlorethylene (PCE), trichlorethylene (TCE), pentachlorobenzene, and carbon tetrachloride [5].

Using HCB as a fungicide was banned by the European Union in 1981, and its production and use was phased out in stages in accordance with the Stockholm Convention signed on 23 May 2001 by 122 countries [6]. HCB is on the list of persistent organic pollutants (POPs) as one of the twelve most dangerous compounds [7]. As a result of its physicochemical properties, resistance to degradation, and tendency to bioaccumulate, this compound spreads and persists in the environment relatively easily [2,3]. Its lipophilic nature means that it easily penetrates the tissues of living organisms and accumulates most often in tissues with high fat contents (Table 1) [8,9].

It is estimated that 88% of the total HCB content in the environment is accumulated in the soil, 9% in the air, 2% in sediments, and 1% in water [2]. Industrial activities probably contributed to increased HCB emissions in the 1990–2014 period [10].

The estimated half-life of HCB in soil is from 2.7 to 22.9 years, in air up to two years, and in water up to six years, which, combined with its chemical properties (poor solubility in water, high stability, high partition coefficient K_OW_ = 5.73), facilitates its movement over considerable distances. HCB biomagnifies as it is passed along in food to subsequent organisms in the food chain. The result of HCB accumulation in the environment is its presence in living organisms [8]. In animals and humans, HCB accumulates in lipid-rich tissues, such as adipose tissue, the adrenal cortex, bone marrow, skin, and some endocrine tissues, and it can be transferred to offspring through both the placenta and breast milk [8].

HCB accumulates in living organisms because it degrades very slowly, and its lipophilic properties permit it to penetrate cell membranes easily [9]. By damaging mitochondria, HCB changes energy dependence from aerobic to anaerobic glycolysis. It can cause porphyria and damage the intestines, kidneys, liver, and brain in animals and humans. HCB can also alter metabolic functions [1]. The liver and reproductive organs are the most susceptible to the effects of HCB [7]. HCB can also irritate the eyes and the respiratory tract and cause skin lesions, photosensitivity, neuroinflammation, and increased porphyrin excretion [2]. As a result of the health hazards of HCB, it is classified as a substance possibly carcinogenic to humans (group 2 B) [11,12].

According to Chen et al. [13], HCB is metabolized into three main metabolites that can be detected in urine: pentachlorophenol (PCP), tetrachlorohydroquinone (TCHQ), and pentachlorothiophenol (PCTP). PCP has been reported to be immunosuppressive in rodents and humans. TCHQ is a major metabolite of PCP and more toxic than PCP. TCHQ has been identified as the main cause of PCP-induced genotoxicity due to reactive oxidant stress (ROS) [13].

Research to date has focused mainly on the possibility of HCB degradation by soil microorganisms for the clean-up of soil contaminated with POPs. This possibility is important for limiting the accumulation in crops of HCB and other toxic compounds from the soil.

Takagi [14] and Ito [15] reported the encouraging discovery of novel aerobic bacteria that degrade POPs (including HCB), novel metabolites, and dehalogenase genes. Takagi [14] described dehalogenase genes associated with the aerobic degradation pathways of HCB by the bacterium strain *Nocardioides* sp. PD653. The proposed degradation pathway is: HCB—PCP (pentachlorophenol)—TeCH (2,3,5,6-tetrachloro-p-hydroquinone)—TCBQ (2,3,5,6-tetrachloro-p-benzoquinone)—TCHQ (2, 5,6-trichloro-p-hydroquinone)—DiCH (2,6-dichloro-p-hydroquinone)—Cl^−^, CO_2_ [14].

Nowadays, trace, but quantitatively detectable, concentrations of HCB are still recorded in the environment, living organisms, and food, and these may pose a threat to consumers because of the durability and toxicity of this compound. The main source of HCB for humans is through dietary ingestion. HCB is found in various amounts in meat, fish [16,17], and dairy products [18,19]. HCB can also accumulate in breast milk. HCB moves easily from the blood of pregnant mothers, across the placenta, to the unborn child and into the breast milk of nursing mothers, resulting in exposure to babies. Since HCB accumulates in fat, the risk for babies (particularly breast-fed infants) may be higher than it is in mothers.

According to Perelló et al. [20], the highest mean HCB levels in food were detected in oils and fats (0.297 ng/g fw), dairy products (0.225 ng/g fw), and fish and seafood (0.170 ng/g fw). As reported by Heck et al. [21], average HCB contents in milk in Brazil ranged from 0.28 to 7.22 ng/g fat. The EFSA reports [22] that HCB, along with DDT and lindane, was one of the insoluble POP pesticides that was most frequently quantified. The maximum permissible content of HCB in milk, regardless of the animal it comes from, expressed as the maximum residue level (MRL), is 0.005 µg/g (Commission Regulation EU 2016/1866) [23].

The literature on this subject states that milk and milk products can be contaminated with a wide range of potentially harmful chemicals that enter the milk by direct and/or indirect routes [24]. Knowledge of the negative effects of toxic substances on health has prompted research into strategies to eliminate, inactivate, or reduce the bioavailability of these compounds in contaminated products [25]. Decontamination procedures must not adversely affect the nutritional value of the food and should be easy to apply [26]. The use of microorganisms for this purpose is justified for food safety and environmental reasons, especially since starter strains are also technical and complementary microflora in fermented milk production [27,28]. The use of probiotic bacteria, which have a positive effect on gut microbiota and are capable of metabolizing contaminants, can be doubly beneficial. Their widespread use in fermented milk raises no nutritional objections, and awareness of their positive effects on human health has already become firmly established in the minds of consumers. The properties of fermented milk containing probiotics have made these products, which are obtained from different types of milk, an important part of the daily human diet. Lactic acid fermentation is the oldest and most widely used method to improve the safety and nutritional value of foods [29]. Recently, many scientific studies have focused on the role of lactic acid bacteria (LAB) in food detoxification [30,31,32]. Live and dead lactobacilli are shown to participate in several health-promoting processes, including food detoxification by adsorbing toxic elements on their cell walls. These processes are thought to involve physical adsorption rather than chemical covalent binding metabolism [33]. It has been observed that the ability of dead lactobacilli is so important in decontamination processes because the viability of the bacteria can be linked to the reduction in low pH conditions in the stomach [34]. The ability of microorganisms to bind is a species- and even strain-specific property of microorganisms [35].

A review of the literature provides us with information on evaluating the ability of *Lactiplantibacillus plantarum* and *Lacticaseibacillus rhamnosus* strains to degrade pesticides and aflatoxins and inhibit the growth of contaminating bacteria, but there was no information on their potential ability to reduce the HCB content in fermented milk [29,36,37]. Unfortunately, allergies to cow milk and its components are becoming increasingly common [38]. These increasing numbers of allergies in children and adults has prompted the search for substitutes that do not cause immune responses [39].

Mare’s milk, which is an excellent alternative to cow’s milk, is gaining consumers gradually, especially in Western Europe (Belgium, France, Austria, Germany, and Italy) and the United States [40]. Barreto et al. [41] reported that approximately 30 million people, mainly in Western Europe and Central Asia, consume mare milk. The promotion of and education about the health-promoting properties of mare milk is increasing consumer demand in many countries, including Poland [42]. This raw material exhibits very high biological activity that results from the presence of lysozyme, lactoferrin, immunoglobulins, and many other bacteriostatic substances [43,44]. The chemical composition of mare milk is significantly different from that of the milk of other animal species. One of its characteristic features is a high lactose content (6.37%) with low fat (1.21%) and protein (2.14%) contents [44,45].

In terms of salt, lactose, and protein content, mare milk is similar to human milk. The fat in mare milk is dispersed in minute globules (approximately 2–3 µm) that are difficult to combine together, which makes separating cream from mare milk almost impossible [46]. The mineral content is almost twice as high as that in human milk (0.42% and 0.22%, respectively) and twice as low as that in cow milk (0.76%) [44,47]. Whey proteins in mare milk constitute 40% of all proteins, as compared to 50% in human milk and 20% in cow milk. As a result of its low casein content of slightly less than 50% of the total protein, it is considered an albumin-type milk, while cow milk is classified as a casein-type milk [48,49]. Mare milk contains little fat, making it easily digestible. The share of saturated fatty acids in mare milk fat is on average 55%, which is less than that in cow milk, while the level of unsaturated fatty acids (44%) is comparable to that in human milk (45.2%) and much higher than that in cow milk (32%) [46]. Mare milk is easily digestible and very well absorbed, and it is suitable for the production of fermented beverages [50].

Prompted by the preceding, the authors decided to address the issue of increasing the health safety of selected dairy products fermented from mare milk by examining the influence selected probiotic bacteria stains have on the biodegradation of carcinogenic HCB.

It was hypothesized that the probiotic strains *Lacticaseibacillus rhamnosus* LCR (Lactoferm LCR Pro-Tek^®^) and *Lactiplantibacillus plantarum* subsp. *plantarum* LP (Lactoferm LP Pro-Tek^®^) (Biochem S.r.l., Monterotondo, Roma, Italy) can effect changes in the HCB content in fermented mare milk. It was also hypothesized that the duration of refrigerated storage might influence the degree of these changes. The aim of the study was to analyze the influence of the probiotic cultures *L. rhamnosus* and *L. plantarum* on changes in the HCB content in fermented mare milk stored under refrigerated conditions (5 ± 1 °C) for 21 days.

## 2. Results

### 2.1. pH and Dry Matter Content in Fermented Mare Milk

The dry matter content in the fermented milk ranged from 8.44 to 9.76% (Table 2), and changes in this during storage were statistically insignificant (*p* > 0.05). There was no significant effect of HCB on the dry matter content of the fermented milk. The highest pH on day 1 of the study period was noted in the Mix_HCB_ sample (4.64), while the lowest was noted in the Mix and LP_HCB_ samples (4.58). During storage, an overall decrease in pH was noted in all variants of the study samples. The greatest change in pH was noted in the Mix sample, while the lowest was in the Mix_HCB_ sample. On the final day of storage, the lowest active acidity was noted in the Mix sample (4.51 pH), and the highest was in the LC sample (4.44 pH). The changes observed during storage were statistically significant (*p* > 0.05). The analysis of the results indicated that the cultures and added HCB did not significantly influence the active acidity of the fermented mare milk beverages.

The mare milk, which was used as a raw material for the production of the fermented beverages, had a dry matter content of 9.4% and fat content of 1.15% at pH 6.46.

### 2.2. HCB in Fermented Mare Milk Beverages

The results indicated that the mare milk used to produce fermented beverages had a low HCB content at an average of 0.09 ± 0.03 ng/mL. To observe changes during refrigerated storage, it was necessary to prepare beverages with added HCB (Table 3). To eliminate error and correctly determine the degree of HCB degradation during refrigerated storage, for the remainder of the experiment, HCB residues in samples to which no HCB was added were subtracted from the values obtained for samples to which HCB was added (Table 3). This permitted calculating the sample enrichment recovery and then calculating the percentage of HCB reduction in subsequent periods of refrigerated storage of the beverages tested.

### 2.3. Changes in HCB Content in Fermented Mare Milk Beverages

The HCB content in the beverages fermented with *L. plantarum* and *L. rhamnosus* and the Mix groups changed during refrigerated storage in all the variants tested (Table 3). Statistical analysis revealed a strong negative correlation (*p* < 0.05) between HCB concentration in fermented mare milk beverages and the period of refrigerated storage. Pearson’s correlation coefficients r were: −0.998 for group C; −0.981 for LP; −0.978 for LC; and −0.975 for Mix. This indicated that the HCB concentrations in the beverages decreased the longer the storage period was (Table 3).

Tukey’s test indicated significant (*p* < 0.05) decreases in HCB content in all analyzed fermented beverages during refrigerated storage; however, after days 14 and 21, these changes were the greatest (*p* < 0.05) (Table 3).

Ultimately, after day 21 of storage, the greatest degree of HCB degradation was confirmed in the beverages that had been fermented with both bacterial strains (Mix groups) with a reduction of 78.77% and with *L. rhamnosus* (78.2%) (Table 3), while the lowest decreases were noted with the application of *L. plantarum* (73.79%).

## 3. Discussion

Between 1990 and 2018, HCB emissions decreased by only approximately 4% to 3707 kg [51]. Changes in HCB emissions resulted from a reduction in emissions from the thermal processing of waste and from small combustion sources in the municipal sector (low emissions) and from an increase in emissions from fuel combustion in power plants and industrial processes (mainly from the production of secondary copper) and from road transport. Considering the scale of the problem, the properties of HCB, and possible detrimental health effects from HCB, including hepatic porphyria, altered thyroid hormones, and tumorigenicity, degradation methods for this highly persistent compound are discussed in the literature, but most research has focused on environmental residues of this pesticide in the air, water, soil, and wastewater [51].

Yan et al. [52] reported that the bacterial culture *Dehalococcoides* sp. CBDB1 and several other mixed cultures in an anaerobic environment led to the bacterial biodegradation and dechlorination of HCB to less chlorinated benzenes (e.g., 1,3,5-trichlorobenzene and 1,2-,1,3-,1,4-dichlorobenzene). They also reported that the resulting products could lead to secondary environmental contamination since they do not degrade further. However, Takagi et al. [14] reported that the bacterium *Neocardina* sp. PD653 can mineralize HCB in aerobic conditions. This bacterium grows by using this pesticide as a nutrient and ultimately degrades this compound into CO_2_ and Cl^−^. This strain is one of the first known natural bacteria that is able to mineralize HCB in aerobic conditions, and it has also proven effective in the bioaugmentation of soils contaminated with POPs. Ji et al. [3] proposed a mechanism for the anaerobic degradation of HCB. Matheus et al. [53] reported on basidiomycetes fungi that are capable of biodegrading organic compounds in contaminated soil and the use of these organisms to recultivate contaminated soils. Takagi et al. [14] proposed a soil and charcoal perfusion method that uses the porous structure of woody material as a habitat for bacteria that decompose HCB; for this, they selected a special strain of bacteria of the genus *Nocardioides* that can dechlorinate HCB from contaminated soil. Currently, the most popular way to minimize the amount of toxic compounds released into the environment is to select appropriate raw materials during production and to employ appropriate technologies. For drinking water production, an ad hoc solution is to use scrubbers and capture filters in treatment plants. For example, Cybulski et al. [54] showed that the treatment process used at the wastewater treatment plant in Szczecin reduced HCB levels by an average of 76.5%.

To provide consumers with food of the lowest possible level of pollutants, it is important to estimate the influence of technological processes in milk and dairy product manufacture on changes in the contents of toxic compounds. The huge interest of consumers in fermented milk products prompted the authors to take up the topic discussed in this work. Previous research by the authors indicated the possibility of obtaining a positive effect, i.e., the reduction in some POPs, such as PCB congeners [55] or selected organochlorine pesticides [56]. The authors proved that the presence of two additional bacterial strains—*Lactobacillus acidophilus* and *Bifidobacterium* sp.—in the A.B.T. bioyogurt starter culture was the likely reason of the high efficiency of this culture in reducing the value of the toxicity equivalent (TEQ_PCB_) in yogurt by nearly 50% [55]. A significantly greater decrease in pesticides was also detected in probiotic beverages prepared from the mixture of two monocultures (*Lb. acidophilus* LA-5 and *Bifidobacterium* BB-12) than in beverages containing only LA-5 [56]. Miśniakiewicz [57] evaluated the effect of LAB on changes in the content of individual dough contaminants and the bread made from it. Rye dough samples were analyzed for organochlorine pesticide residues (lindane, aldrin, dieldrin, o,p-DDT, p,p-DDT, o,p-methoxychlor, p,p-methoxychlor, and the pesticide metabolite 3,5-dichloroaniline were determined). In the dough tested, 3,5-dichloroaniline and endrin were found, and the residues of organochlorine pesticides detected were reduced by fermentation.

It is difficult to find information in the literature on the role of lactobacilli in reducing HCB concentrations in food, but there are papers on the use of these cultures in other detoxification processes. Liu et al. [58] showed that LAB can be an effective alternative in bio-detoxification. A proposed mechanism of detoxification is the adsorption of heavy metals and other toxins by the cell walls of certain LAB strains [33]. The authors investigated the possibility of using a single strain or a combination of LAB to remove heavy metals (arsenic, cadmium, chromium, copper, and lead), cyanotoxins (microcystin-LR, -RR, and -LF) and mycotoxins (aflatoxin B1, B2, B2 a, M1, M2, G1, G2, patulin, ochratoxin A, deoxynivalenol, fumonisin B1 and B2, 3-acetyldeoxynivalenol, deoxynivalenol, fusarenone, nivalenol, diacetoxyscirpenol, HT-2 and T-2 toxin, and zearalenone and its derivatives) from aqueous solutions in vitro. Wang et al. [59] and Gerbino et al. [60] reported that LAB are able to detoxify heavy metals. Similarly, Ninkov et al. [61] reported that lactobacilli have the ability to convert methylated Hg into the less toxic inorganic form that is not absorbed in the gastrointestinal tract. Based on the review of the literature, the mechanism of heavy metal detoxification by *Lactobacillus* ssp. is explained by binding metal ions through the cell wall and bioaccumulation in bacterial cells. The surface of the microorganisms has a negative charge at neutral pH, so it is able to bind with the cationic form of heavy metals [62,63]. The detoxifying capacity of LAB depends on the pH value and the concentration and specificity of the strain [64], the acidity of the environment, the temperature, and the initial concentration of the toxins. There is no proportional relationship between temperature and the degree of detoxification. Different species show different efficiencies in this type of process depending on temperature [65]. When considering the effect of pH, it can be seen that in general, low bio-adsorption was reported at pH ranges below 3, but with increasing pH and above pH 3, a sharp increase in removal was observed that reached maximum values at pH 6 [63]. Some organic acids have also been reported to increase the amount of bio-adsorption of heavy metals, possibly because of the effect of pH [66,67]. Exopolysaccharides produced by LAB may play a role in detoxification [68,69]. Since they differ in structure, binding capacity, and chemical composition, their mechanism of action is not fully understood.

The current study confirmed that it is also possible to reduce HCB content using bacteria in products intended for consumption, such as fermented beverages. The reduction in HCB that was achieved was not total, but ranged from 73.8 to 78.8%.

## 4. Materials and Methods

### 4.1. Study Material

The material for the study was fermented mare milk produced by the thermostat method under laboratory conditions [70,71]. The milk (9.4% dry matter content, pH 6.46, density 1.034 g/mL, 1.15% fat content) was purchased from a horse dairy farm in Kłodzin (Poland). After the milk was transported under refrigeration to the laboratory, it was pasteurized (vat method, 85 ± 1 °C, 30 min) and then cooled to a temperature of 42 ± 1 °C [72]. Milk for the production of fermented milk with monocultures was pasteurized with the long and high method (batch pasteurization), the parameters of which depend on the dry matter content; typically, temperatures in the range of 85–95 °C are applied for 5–30 min. The aim of pasteurization is to destroy milk microflora and to inactivate enzymes, which provide good conditions for starter culture development. Another aim is the denaturation of whey protein and their interaction with casein micelles, which prevents possible defects in structure, including separation and low curd viscosity [73].

The cooled milk (42 ± 1 °C) was divided into two parts, and a standard solution (HCB solution, certified reference material; Supelco 40008, Darmstadt, Germany) containing a known amount of HCB (93.5 ng/mL) was added to one part to permit identifying and determining changes in the content of this compound. After HCB was added to the sample, the material was homogenized. The second part of the milk was used to prepare control samples without HCB. Both parts of the milk were inoculated with pre-activated bacterial cultures at 7% (*v*/*v*). The inoculum was obtained by incubating a portion of culture (0.6 g/L) in skimmed milk (0.0%) for 4–8 h at 40 °C. The end of fermentation was determined based on the pH and fermentation curve set in the culture specification. The following concentrated, direct vat inoculation (DVI) freeze-dried probiotic starter cultures were used in the experiment: *Lactiplantibacillus plantarum* LP (Lactoferm LP Pro Tek^®^; Biochem s.r.l., Italy, containing the activated form of inoculum 1.62∙10^9^ CFU/g) and *Lacticaseibacillus rhamnosus* LC (Lactoferm LCR Pro Tek^®^; Biochem s.r.l., Italy, containing the activated form of inoculum 1.54∙10^9^ CFU/g). All sample variants (with and without HCB added) were poured into 50 mL containers, hermetically sealed, coded, and placed in an incubation chamber for fermentation. Ultimately, six variants of samples were prepared (3 with and 3 without HCB). Fermentation was conducted at 35 ± 1 °C for 4–8 h to obtain pH 4.65–4.60. The samples were then stored under refrigeration (5 ± 1 °C) for 21 days. The fermented milk variants were prepared as presented in Table 4.

Samples were collected randomly at seven-day intervals, on days 1, 7, 14, and 21 of storage, to determine the dry matter and pH, and then these samples were frozen (−21 ± 1 °C) for further analyses.

### 4.2. Analysis of pH and Dry Matter Content of Fermented Milk

The pH of the fermented milk was measured with a pH meter (Milwaukee MW101 PRO, Milwaukee Instruments, Inc., Rocky Mount, NC, USA) before freezing the samples. The dry matter content was determined with the oven-drying gravimetric method [74]. Determinations were performed three times on each sample.

### 4.3. Analysis of HCB Content

Prior to analysis, samples were freeze-dried in a LyoLab 3000 (Fisher Scientific, Loughborough, Leicestershire, UK England) at lowered pressure and at a temperature of −60 ± 2 °C; then, after securing the samples, they were stored at a temperature of −21 ± 1 °C.

**Figure 1:**
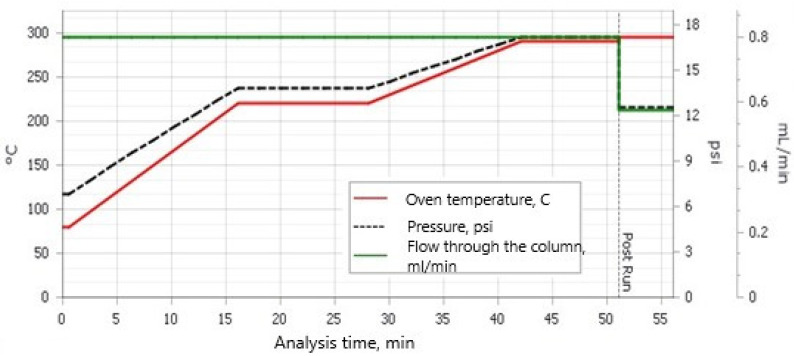
Chromatographic separation conditions.

The samples were extracted in a Soxhlet extractor with 150 mL of a solvent mixture of hexane/acetone (*v*/*v*) (3/1) for 8 h. Then, the samples were concentrated in an R-300 rotary vacuum evaporator (Büchi Rotavapor R-300 with Büchi Heating Bath B-300 Base; BÜCHI Labortechnik AG, Flawil, Switzerland) to approximately 2 mL, quantitatively transferred to 10 mL test tubes, and then concentrated to approximately 2 mL in a stream of nitrogen. In the next stage, the samples were cleaned with concentrated H_2_SO_4_, and then further cleaned in LiChlorut glass columns (Merck, Darmstadt, Germany) on a bed of 1.5 g florisil, and finally concentrated to 0.5 mL in a stream of nitrogen. Quantitative analysis was performed on a GC/MS device (Agilent 8890, Mass Selective Detector HP 5977; Agilent, Santa Clara, CA 95051, USA) with a HP 5 MS column (30 m × 250 µm × 0.25 µm). The carrier gas was helium 6.0, and each sample injection was 2 μL. The chromatographic separation conditions are presented in Figure 1.

**Figure 2:**
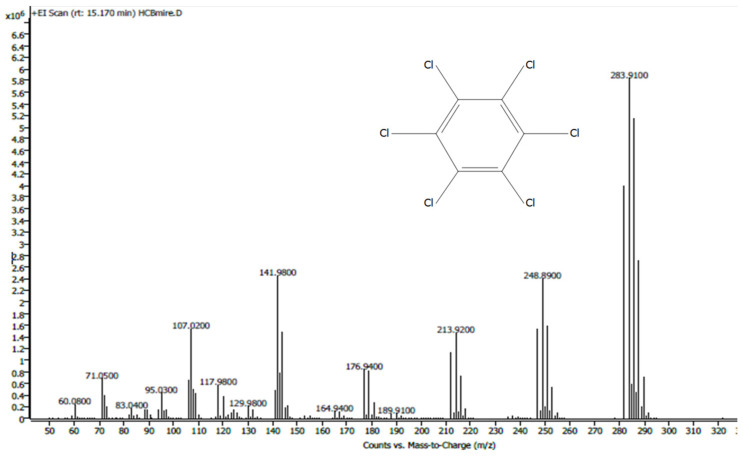
HCB mass spectrum.

HCB was determined with a mass spectrum histogram (analysis in SCAN MS mode; Figure 2). To quantify the pesticide, the actual samples and calibration standard solutions were applied in the Selected Ion Monitoring (SIM) mode of the mass spectrometer (target ion [*m*/*z*] 283.9; confirmation ions: 248.9; 141.9). Each sample was analyzed three times.

### 4.4. Statistical Analysis

The results were analyzed statistically with Statistica 13.3. ANOVA (analysis of variance) was preceded by the Levene’s homogeneity test and the Kolmogorov–Smirnov normal distribution test (K–S test). Pearson correlation coefficients were also determined. The significance of differences among mean values was evaluated with Tukey’s test (*p* < 0.05).

## 5. Conclusions

The current study confirmed that it is possible to reduce HCB content using bacteria, not only in the environment (waters, soils, and air), but also in products intended for consumption.

Mare milk is becoming increasingly popular thanks to, among other things, the growing trend in following a healthy lifestyle and diet. Appreciation for food products with properties such as those in mare milk is growing, and not just for the milk itself, but also other forms including powdered or encapsulated milk that simplify storage and use. Since HCB is ubiquitous in the environment, highly persistent, and bioaccumulates in the human body, it poses risks in the form of residues in mare milk, a valuable raw material. Similarly to other POPs, it is difficult to eliminate completely, but steps should be taken to reduce the content of HCB to minimal levels that pose no threat to consumers. Bearing in mind bioaccumulation, consuming even trace amounts over long periods of time could have consequences that are difficult to evaluate. This is particularly true in the case of infants, for whom mare milk is recommended. This is why research must be conducted on eliminating HCB not only from the environment, but especially from products intended for consumption.

The current study indicated that the type of bacterial strain used influenced the degree of HCB reduction during the storage of fermented beverages made with mare milk, but the differences among all variants were not large. The highest HCB reduction was noted after day 21 of storage in milk fermented with a mix of the two probiotic cultures of *L. rhamnosus* and *L. plantarum* (78.77%), while the lowest reduction was noted when only *L. plantarum* (73.79%) was used.

The current study confirmed that the bacterial cultures used are an alternative method for reducing HCB residues in fermented beverages.

## Figures and Tables

**Table 1:** Physicochemical properties of HCB.

Summary Formula	Structural Formula	Molar Mass (g/mol)	Melting Point (°C)	Boiling Point (°C)	Solubility in Water (mg/L)	Log K_OW_ *	Bioconcentration Factor (BCF)
C_6_Cl_6_	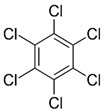	284.78	231.0	322	0.006	5.73	35,000

* K_OW_—octanol–water partition coefficient.

**Table 2:** Influence of refrigerated storage on the pH and dry matter content of fermented mare milk with and without added HCB.

Sample Variant		Storage Time (Days)			
		1	7	14	21
		pH ^1^			
without added HCB	LP	4.63 ± 0.01 ^aA^	4.63 ± 0.01 ^aA^	4.50 ± 0.01 ^bA^	4.48 ± 0.02 ^cA^
	LC	4.60 ± 0.02 ^aA^	4.60 ± 0.01 ^aA^	4.47 ± 0.02 ^bA^	4.44 ± 0.01 ^cA^
	Mix	4.58 ± 0.02 ^aA^	4.54 ± 0.01 ^bA^	4.52 ± 0.01 ^cA^	4.51 ± 0.01 ^dA^
with added HCB	LP_HCB_	4.58 ± 0.02 ^aA^	4.53 ± 0.01 ^bA^	4.50 ± 0.01 ^cA^	4.48 ± 0.01 ^dA^
	LC_HCB_	4.60 ± 0.01 ^aA^	4.58 ± 0.01 ^bA^	4.30 ± 0.01 ^dA^	4.47 ± 0.02 ^cA^
	Mix_HCB_	4.64 ± 0.02 ^aA^	4.62 ± 0.01 ^bA^	4.30 ± 0.02 ^dA^	4.47 ± 0.01 ^cA^
		**Dry weight (%) ^1^**			
without added HCB	LP	9.11 ± 0.01 ^aA^	9.10 ± 0.01 ^aA^	9.11 ± 0.01 ^aA^	9.13 ± 0.01 ^aA^
	LC	8.79 ± 0.02 ^aA^	8.82 ± 0.01 ^aA^	8.80 ± 0.01 ^aA^	8.79 ± 0.01 ^aA^
	Mix	9.56 ± 0.02 ^aA^	9.51 ± 0.01 ^aA^	9.52 ± 0.01 ^aA^	9.56 ± 0.01 ^aA^
with added HCB	LP_HCB_	9.62 ± 0.01 ^aA^	9.62 ± 0.02 ^aA^	9.62 ± 0.01 ^aA^	9.62 ± 0.01 ^aA^
	LC_HCB_	8.44 ± 0.02 ^aA^	8.44 ± 0.01 ^aA^	8.44 ± 0.02 ^aA^	8.44 ± 0.02 ^aA^
	Mix_HCB_	9.52 ± 0.02 ^aA^	9.54 ± 0.02 ^aA^	9.49 ± 0.02 ^aA^	9.51 ± 0.02 ^aA^

^1^ arithmetic mean ± standard deviation; lowercase letters—significant differences (*p* < 0.05) during storage in individual variants of fermented beverages; capital letters—significant differences (*p* < 0.05) between samples inoculated with the same strain (pair-wise comparison samples with and without HCB).

**Table 3:** Changes in HCB content in fermented mare milk beverages during refrigerated storage.

Refrigerated Storage Period	Sample Variant		HCB Content in Beverages to Which HCB Was Not Added	HCB Content in Beverages to Which HCB Was Added (93.5 ng/mL)	Recovery of Added HCB, ng/mL	Average HCB Reduction, %
		HCB Concentration, ng/mL				
day 1	*L. plantarum* (LP)	x ^1^	0.099 ^bB^	80.83 ^aB^	80.73	13.67%
		SD ^2^	0.0029	1.144	1.499	
		Me ^3^	0.099	80.18	80.71	
		CV ^4^	2.89	1.415	1.86	
	*L. rhamnosus* (LC)	x	0.144 ^bA^	82.31 ^aA^	82.16	12.13%
		SD	0.0025	1.426	1.712	
		Me	0.145	82.29	81.29	
		CV	1.73	1.732	2.09	
	Mix	x	0.113 ^bB^	83.44 ^aA^	83.33	10.88%
		SD	0.003	1.892	3.398	
		Me	0.114	82.88	82.00	
		CV	2.20	2.27	4.08	
day 7	*L. plantarum* (LP)	x	0.090 ^bB^	73.72 ^bB^	73.63	21.25%
		SD	0.006	2.117	2.732	
		Me	0.089	72.64	73.96	
		CV	6.88	2.87	3.71	
	*L. rhamnosus* (LC)	x	0.121 ^bA^	76.67 ^bA^	70.55	24.55%
		SD	0.0059	2.676	3.371	
		Me	0.119	75.83	71.02	
		CV	4.87	3.49	4.78	
	Mix	x	0.107 ^bA^	78.79 ^bA^	78.68	15.85%
		SD	0.0062	1.769	2.617	
		Me	0.108	78.90	80.00	
		CV	5.76	2.25	3.33	
day 14	*L. plantarum* (LP)	x	0.056 ^cA^	50.94 ^cA^	50.89	45.57%
		SD	0.0037	1.821	2.831	
		Me	0.055	51.29	51.79	
		CV	6.68	3.58	3.56	
	*L. rhamnosus* (LC)	x	0.039 ^cB^	44.48 ^cB^	44.44	52.47%
		SD	0.003	2.365	4.118	
		Me	0.0372	43.81	45.11	
		CV	9.49	5.32	9.27	
	Mix	x	0.032 ^cB^	44.75 ^cB^	44.72	52.17%
		SD	0.0036	3.695	0.880	
		Me	0.030	45.04	44.25	
		CV	11.12	8.26	1.97	
day 21	*L. plantarum* (LP)	x	0.061 ^dA^	24.57 ^dA^	24.51	73.79%
		SD	0.0016	1.919	1.693	
		Me	0.061	25.04	24.29	
		CV	2.68	7.81	6.87	
	*L. rhamnosus* (LC)	x	0.039 ^dB^	20.41 ^dB^	20.37	78.22%
		SD	0.0030	1.744	1.125	
		Me	0.038	20.21	20.57	
		CV	7.55	8.55	5.53	
	Mix	x	0.014 ^dC^	19.86 ^dB^	19.85	78.77%
		SD	0.0014	1.590	0.803	
		Me	0.013	20.47	20.14	
		CV	10.10	8.01	4.04	

^1^ X—arithmetic mean; ^2^ SD—standard deviation; ^3^ Me—median; ^4^ CV—coefficient of variation, %; lowercase letters—significant differences (*p* < 0.05) during storage in individual variants of fermented beverages; capital letters—significant differences (*p* < 0.05) between variants of beverages fermented at one storage.

**Table 4:** Experimental variants of fermented mare milk.

Sample Code		Sample Description
Beverages without added HCB	LP	Mare milk fermented with a culture of *Lactiplantibacillus plantarum*
	LC	Mare milk fermented with a culture of *Lacticaseibacillus rhamnosus*
	Mix	Mare milk fermented with a mixed culture of *L. plantarum* and *Lacticaseibacillus rhamnosus* at a ratio of 1:1
Beverages with added HCB	LP_HCB_	Mare milk with added HCB fermented with a culture of *Lactiplantibacillus plantarum*
	LC_HCB_	Mare milk with added HCB fermented with a culture of *Lacticaseibacillus rhamnosus*
	Mix_HCB_	Mare milk with added HCB fermented with a mixed culture of *Lactiplantibacillus plantarum* and *Lacticaseibacillus rhamnosus* at a ratio of 1:1

## Data Availability

Data are contained within the article.
